# Risk of Cancers in Antineutrophil Cytoplasmic Antibody-Associated Vasculitis: Results from the Korea National Health Insurance Claims Database 2010–2018

**DOI:** 10.3390/jcm8111871

**Published:** 2019-11-05

**Authors:** Sung Soo Ahn, Minkyung Han, Juyoung Yoo, Seung Min Jung, Jason Jungsik Song, Yong-Beom Park, Inkyung Jung, Sang-Won Lee

**Affiliations:** 1 Division of Rheumatology, Department of Internal Medicine, Yonsei University College of Medicine, Seoul 03722, Korea; saneth@yuhs.ac (S.S.A.); juyoung@yuhs.ac (J.Y.); jsmin00@yuhs.ac (S.M.J.); jsksong@yuhs.ac (J.J.S.); yongbpark@yuhs.ac (Y.-B.P.); 2 Biostatistics Collaboration Unit, Department of Biomedical Systems Informatics, Yonsei University College of Medicine, Seoul 03722, Korea; minkyunghan@yuhs.ac; 3 Institute for Immunology and Immunological Diseases, Yonsei University College of Medicine, Seoul 03722, Korea; 4 Division of Biostatistics, Department of Biomedical Systems Informatics, Yonsei University College of Medicine, Seoul 03722, Korea

**Keywords:** antineutrophil cytoplasmic antibody, vasculitis, cancer, incidence, risk

## Abstract

The association between antineutrophil cytoplasmic antibody-associated vasculitis (AAV) and cancer remains poorly understood. In this study, we searched the Korea National Health Insurance Claims Database to obtain data for 2097 AAV patients, and evaluated the risk of cancers in AAV. The standardized incidence ratios (SIRs) of overall and site-specific cancers were estimated in patients with AAV compared to the general population. The overall risk of cancer was significantly higher in patients with AAV (SIR 1.90); this remained true in both males (SIR 1.74) and females (SIR 2.06). For site-specific cancers, the risks of lung (SIR 2.23) and hematological (SIR 11.39) cancers were higher in AAV patients. For males, the risks of gallbladder and hematological cancers were increased, while the risks of bladder and hematological cancers were increased in females. Among AAV subtypes, patients with granulomatosis with polyangiitis had the highest risk of cancers, and cyclophosphamide, azathioprine/mizoribine, and methotrexate ever-users had increased risk of overall cancer. The risks of overall and hematological cancers were elevated in AAV patients younger than 60 years old. Patients with AAV have increased risks of overall, lung, and hematological cancers. Distinct patterns of cancer incidence are present according to age, sex, AAV subtypes, and immunosuppressant usage.

## 1. Introduction

Antineutrophil cytoplasmic antibody (ANCA)-associated vasculitis (AAV) is a rare autoimmune disease (AID) with the hallmarks of chronic inflammation within the blood vessels and the production of ANCAs directed against myeloperoxidase (MPO) and proteinase 3 (PR3) [1]. AAV is classified into three different disease entities based on the characteristic histologic, serologic, and clinical features [2]. Epidemiologic studies have indicated that AAV mainly affects patients over 50 years old, with an estimated incidence of 10–20/million and a prevalence of over 100/million [3,4,5]. Various comorbidities, such as cancers, infections, cardiovascular disease, and osteoporosis, are prevalent in patients with AIDs [6,7,8]. Among the comorbid conditions, the relationship between AIDs and cancers has attracted special attention because subjects with AIDs are at higher risk of developing cancers, and the prognosis of such patients is particularly unfavorable [9]. In addition, there is evidence that cancer can essentially trigger the development of autoimmunity by affecting the immune system, suggesting a ‘bi-directional’ relationship [10,11]. Meanwhile, the long-term administration of immunosuppressive agents that are used for the treatment of AIDs is also believed to support the development of cancers [12,13]. For this reason, continuous efforts have been made to explore the link between AIDs and cancers.

Generally, a significantly higher incidence of cancers has been demonstrated in patients with AAV [14]. A meta-analysis that analyzed the risk of cancers in patients with AAV showed a higher overall risk of cancer, specifically non-melanoma skin cancer (NMSC), leukemia, and bladder cancer [15]. Moreover, cyclophosphamide, which is the most widely used therapeutic agent to induce remission in AAV, is thought to influence the development of cancers [16]. However, there are inconsistencies in the risks of site-specific malignancies in AAV. Data from the European Vasculitis Study group have shown that only NMSC was more common in patients with microscopic polyangiitis (MPA) and granulomatosis with polyangiitis (GPA), while the results from the study by Westman et al. [17] revealed a higher risk of bladder, testicular, and skin cancer in MPA and GPA [17,18]. Furthermore, a study by Tatsis et al. suggested that the risk of renal cell carcinoma was elevated in patients with GPA compared to those with rheumatoid arthritis [19]. Notably, clear geographic and ethnic disparities have been reported with regard to the demographic factors affecting the link between AAV and cancer. For instance, although GPA and PR3-ANCA vasculitis are more common in Western countries, MPA and MPO-ANCA vasculitis are more frequent in Asian countries. In addition, males seem to be more frequently affected in Western countries, but female patients with AAV are more common in the Eastern world [20,21]. Moreover, there is considerable variation in the incidence of cancer according to sex, geographic location, and ethnicity [22,23]. Until recently, because of the rarity of the disease and the difficulty in diagnosing AAV, the incidence of cancers in patients with AAV has not been well understood. Therefore, the objective of this study was to evaluate the risk of cancers in Korean patients with AAV by utilizing the Korea National Health Insurance Claims data.

## 2. Materials and Methods 

### 2.1. Definition of Patient and the Source of Data

All data were acquired from the database operated by the Health Insurance and Review Agency (HIRA) in Korea. The HIRA database is a comprehensive database that collects all health care utilization information registered by Korea’s National Health Insurance (NHI), a mandatory, nationwide health insurance system. As such, the HIRA database covers nearly the entire population of Korea, containing information on over 50 million subjects in the country. All medical records are integrated into the HIRA claims database through the electronic submission of health care utilization information by medical institutions via electronic form to the NHI for reimbursement purposes. Briefly, the database includes a wealth of information on all patients, including the following: Hospitalization, ambulatory care, demographic characteristics, principle diagnoses and comorbidities (International Classification of Diseases (ICD)-10 codes), drug prescriptions and procedures. Therefore, by using the HIRA database, it is possible to provide estimates representing the whole spectrum of disease extent and severity in unselected patient populations [24]. 

To limit our population to AAV patients, we enrolled patients who were diagnosed with AAV in a general or tertiary hospital and were prescribed glucocorticoids (methylprednisolone, hydrocortisone, prednisone, prednisolone, triamcinolone, budesonide, betamethasone, dexamethasone, and deflazacort). In the identification of MPA, GPA (which is identical to Wegener’s granulomatosis), and eosinophilic granulomatosis with polyangiitis (EGPA) (which is identical to Churg-Strauss syndrome), the ICD-10 codes M31.7, M31.3, and M30.1 were used. Regarding immunosuppressive agents that were administered to AAV patients after the diagnosis but prior to the development of cancer, the medications of cyclophosphamide, rituximab, azathioprine/mizoribine, and methotrexate were defined as AAV medications. Because methotrexate can also be used in the treatment of cancers, any dose over the standard dose prescribed for the treatment of AAV (>25 mg) was excluded.

The initial date of the registration of the corresponding diagnosis in the HIRA database (index date) was considered the date of AAV diagnosis. The original source of the population for this study consisted of all National Health Insurance Claims data from 2008 to 2018. Furthermore, a washout period of 2 years was applied to exclude the previous prevalent cases. As a result, all the data analyzed were from patients who were diagnosed with AAV between January 2010 and December 2018. This study was approved by the ethics review board of the Hospital, and the need to obtain informed consent was waived, owing to the retrospective study design (IRB approval number: 4-2019-0177).

### 2.2. Cancer Case Ascertainment and Charlson Comorbidity Index

The incidence of cancer in patients with AAV after the diagnosis was investigated from the National Health Insurance Claims Database and from the 2014 National Cancer Registry (NCR) for the general population [25]. The definition of cancer occurrence was set as both hospital admission and the registration of a cancer code (ICD-10: C00–C96) in the National Health Insurance database [26]. In short, all incident cancer cases were defined as those admitted to the hospital for cancer. The medical conditions included in the Charlson Comorbidity Index (CCI), which is an index that categorizes the comorbidities of patients based on the ICD codes found in administrative data, were investigated by searching the corresponding ICD-10 codes of the patients within one year of the AAV index date, as previously described [27].

### 2.3. Statistical Analysis

To compare the incidence of cancer in patients with AAV and the general population, we calculated the standardized incidence ratios (SIRs) of overall and site-specific cancers after dividing patients by age into 10-year intervals. The observed and expected number of cases were calculated for each age group using age and sex-specific incidence data. Site-specific cancers were selected based on the 10 most common cancers in the general population in both males and females, which were included in the 2014 NCR report [25]. Furthermore, in all subgroup analyses, the overall SIR for all types of hematological cancer (which included Hodgkin lymphoma (C81), non-Hodgkin lymphoma (C82–C85, C96), multiple myeloma (C90), and leukemia (C91–C95)), bladder cancer (C67), and other cancers (remaining cancer codes) were also calculated. The SIR was estimated by dividing the number of observed cancers by the number of expected cancers, and the 95% confidence interval (CI) was estimated by the Poisson distribution. We calculated the number of expected cancer cases by multiplying the age-specific cancer incidence rate of the general population from the 2014 NCR and the person-years of patients with AAV.

We investigated factors associated with cancer development among patients with AAV using a nested case-control analysis to avoid length bias. In the nested case-control analysis, age, sex, the index date of AAV diagnosis, duration, and type of diagnosis were matched. Conditional logistic regression was performed to estimate the odds ratio (OR) and corresponding 95% CI. A *p*-value less than 0.05 was considered statistically significant, and SAS Enterprise Guide version 9.4 (SAS Institute Inc., Cary, NC, USA) and R 3.6.1 (R Foundation for Statistical Computing, Vienna, Austria) were used for all statistical analyses.

## 3. Results

### 3.1. Baseline Characteristics of Patients with AAV

In total, 2425 incident cases of AAV were identified during the period from 2010 to 2018. Among the patients recruited, 328 patients were excluded due to a previous history of cancer before the diagnosis of AAV. With regard to the type of cancer prior to the diagnosis of AAV, lung cancer (C33–C34) was the most common, followed by colon and rectum cancer (C18–C20), prostate cancer (C61), and non-Hodgkin lymphoma (C82–C85, C96) (Supplementary Figure S1). Among the selected 2097 patients, the mean age was 59.8 years, and the proportion of female patients was 55.7%. In terms of the subgroups of AAV, 947 (45.2%) patients were diagnosed with MPA, 568 (27.1%) patients were diagnosed with GPA, and 582 (27.7%) patients were diagnosed with EGPA (Table 1). The mean age at diagnosis was 64.3, 58.3, and 53.8 years for patients with MPA, GPA, and EGPA, respectively.

### 3.2. Comparison of Variables between AAV Patients with and without Cancer

A total of 114 patients (5.4%) developed cancer after the diagnosis of AAV, and 1983 (94.6%) patients did not develop cancer during the mean follow-up duration of 3.0 years and 6357.7 person-years (Supplementary Figure S1). The mean follow-up duration for AAV patients was 1.5 years for those with cancer and 3.1 years for those without cancer.

The baseline characteristics of AAV patients with cancer and without cancer are described in Table 1. There were no statistically significant differences regarding the age at diagnosis, sex, type of insurance, and the medical conditions constituting the CCI, but the proportion of GPA patients was higher in AAV patients with cancer (43.0% vs. 26.2%). Regarding medication usage, AAV patients with cancer were more often treated with methotrexate (21.9% vs. 13.5%, *p* = 0.0173)

### 3.3. Estimation of Cancer Risk in AAV Patients According to Sex

When we calculated the SIRs of cancers in AAV patients, it was found that the overall cancer risk was significantly higher than that in the general population (SIR 1.90, 95% CI 1.57–2.28). For site-specific cancers, the risks of lung cancer (C33–C34) (SIR 2.23, 95% CI 1.34–3.48), hematological cancer (SIR 11.39, 95% CI 7.44–16.69), and the remaining cancers (SIR 3.11, 95% CI 1.97–4.66) were increased. Within hematological cancer, the risks of non-Hodgkin lymphoma (C82–C85, C96) (SIR 10.14, 95% CI 5.40–17.34) and multiple myeloma (C90) (SIR 21.12, 95% CI 10.13–38.85) were elevated (Table 2). NMSC (C44) was only found in one female patient, and the risk for NMSC was not significant.

In male patients with AAV, the risk of overall cancer (SIR 1.74, 95% CI 1.33-2.24) was elevated, and for site-specific cancers, the risks of gallbladder (C23–C24) (SIR 3.82, 95% CI 1.04-9.78), lung (C33–C34) (SIR 2.23, 95% CI 1.22–3.74), and hematological cancers (SIR 9.59, 95% CI 4.96–16.76) were higher compared to those in the general population. On the other hand, although the risk of overall cancer was also elevated in female patients with AAV, an increased risk of bladder cancer (C67) (SIR 12.05, 95% CI 2.48–35.21) and an increased risk of the remaining cancers (SIR 4.34, 95% CI 2.37–7.28), but not gallbladder or lung cancers, were observed (Table 3).

### 3.4. Risk of Cancer Based on AAV Subtypes

Next, we evaluated whether there were differences in the risk of cancer according to AAV subtypes. In a subgroup analysis, an increased risk of cancer in comparison to that of the general population was found across the three subtypes of AAV, but the SIR was the highest in patients with GPA (SIR 2.85, 95% CI 2.11–3.77). Concerning site-specific cancers, only hematological cancer (SIR 5.86, 95% CI 2.15–12.75) was found to be elevated in patients with MPA, whereas the risks of lung (C33–C34) (SIR 4.17, 95% CI 2.00–7.66), hematological (SIR 18.40, 95% CI 9.51–32.15), and the remaining cancers (SIR 6.15, 95% CI 3.27–10.51) were higher in GPA patients. Meanwhile, in EGPA patients, the risks of hematological (SIR 13.20, 95% CI 5.70–26.01) and the remaining cancers (SIR 3.13, 95% CI 1.15–6.82) were found to be elevated (Table 4). 

### 3.5. Risk of Cancer Based on Immunosuppressive Agent Usage

We divided our patients into ever- and never-users of cyclophosphamide, rituximab, azathioprine/mizoribine, and methotrexate prior to the diagnosis of cancer and estimated the SIRs for cancer development. As shown in Supplementary Table S1 and S2, the risk for overall cancer was higher in patients treated with immunosuppressants, except in those treated with rituximab. However, patients treated with rituximab had an increased risk of the site-specific kidney cancer (C64) (SIR 15.39, 95% CI 1.86–55.61) and non-Hodgkin lymphoma (C82–C85, C96) (SIR 14.47, 95% CI 1.75–52.26). Patients who were treated with cyclophosphamide had significantly higher risks of lung cancer (C33–C34) (SIR 2.32, 95% CI 1.11–4.27), kidney cancer (C64) (SIR 5.01, 95% CI 1.03–14.63), hematological cancer (SIR 13.82, 95% CI 7.90–22.45), and the remaining cancers (SIR 3.51, 95% CI 1.87–6.00). Patients that were administered with azathioprine/mizoribine had higher risk of kidney cancer (C64) (SIR 5.46, 95% CI 1.13–15.96), hematological cancer (SIR 5.67, 95% CI 2.08–12.35), and the remaining cancers (SIR 3.25, 95% CI 1.62–5.81). Methotrexate users showed an increased risk of hematological cancer (SIR 17.72, 95% CI 6.50–38.57) and remaining cancers (SIR 7.44, 95% CI 3.21–14.66).

In addition, a nested case-control analysis was performed to elucidate whether the use of immunosuppressive agents was associated with the risk of cancers among AAV patients. In the adjusted analysis, the use of glucocorticoid for ≥1 year (OR 4.20, 95% CI 1.47–12.01, *p* = 0.0075), the use of cyclophosphamide (OR 1.75, 95% CI 1.03–2.96, *p* = 0.0385), and the use of methotrexate (OR 2.39, 95% CI 1.12–5.13, *p* = 0.0247) were found to be independently associated with the development of cancers (Table 5).

### 3.6. Overall, Hematological, and Lung Cancer Risks in Patients with AAV According to Age

**Figure 1:**
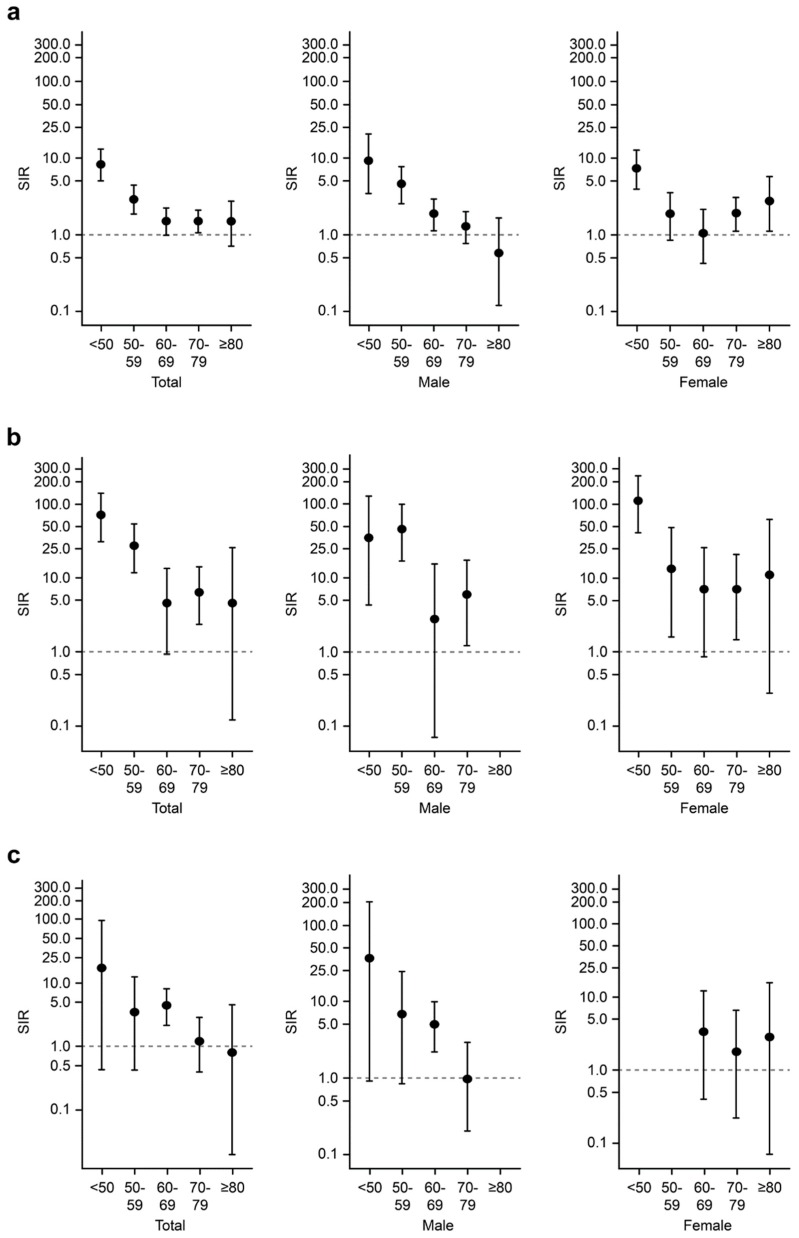
Risks of overall, hematologic, and lung cancers according to age groups. The standardized incidence ratios of (**a**) overall, (**b**) hematologic, and (**c**) lung cancers were assessed in the patients with AAV according to age groups. AAV: ANCA-associated vasculitis; ANCA: Antineutrophil cytoplasmic antibody.

Finally, we evaluated whether there was a difference in the risk of cancer according to age groups. Because the risks for overall cancer, hematological, and lung cancer were significantly increased compared to those in the general population, the SIRs for those three groups were evaluated according to age. Regarding the risk of overall cancer in AAV patients, the risk was higher across all age groups except in those ≥80 years old. However, in males, the risk of cancer was higher in the age groups of <50, 50–59, and 60–69 years old. In contrast, in females, patients <50, 70–79, and ≥80 years old were at increased risk of overall cancers (Figure 1a).

For hematological cancers, the risk was higher in those <50, 50–59, and 70–79 years old among AAV patients, whereas the risk was found to be elevated in those <50, 50–59, and 70–79 years old among males and <50, 50–59, and 70–79 years old among females (Figure 1b). Regarding lung cancer, the risk was only significantly increased in those who were 60–69 years old in AAV patients (Figure 1c).

## 4. Discussion

Although it is now becoming increasingly evident that the risk of cancers is elevated in subjects with AIDs, the temporal relationship between AAV and cancer remains unclear. In the present study, we assessed the risk of cancer in patients with AAV by utilizing the Korea National Health Insurance Claims Database. The observations from our study indicated that among the patients included, a substantial proportion of AAV patients (5.4%) developed cancer during the follow-up period. Overall, patients affected with AAV had a significantly higher risk of developing cancers (SIR 1.90) compared to the general population, which is comparable to results previously reported in the literature [28]. Moreover, regarding site-specific cancers, the risks of lung and hematologic cancers were especially higher compared to those in the general population. Additionally, the diagnosis of GPA and the administration of immunosuppressive agents except rituximab were associated with an increased risk of overall cancer. The findings from our study have important implications in the management of AAV.

In general, chronic inflammation is associated with the risk of cancers by altering the immune response [29], and both systemic and localized inflammation in AIDs may contribute to the development of cancer. The effect of inflammation on tumorigenesis is a complicated process mediated by the release of cytokines, chemokines, and growth factors; the promotion of angiogenesis; and damage to DNA [30,31]. In addition, functional defects are found in the regulatory T cell populations in AIDs, which could lead to cancer progression by the suppression of anti-tumor surveillance [32,33]. In this study, we found that the risks of site-specific lung and hematologic cancers are significantly elevated in patients with AAV. This could be explained by the fact that pulmonary involvement is a common manifestation in AAV and autoimmunity is closely linked to the development of hematologic cancers as a consequence of abnormal cellular lineage expansion and the impaired clearance of autoreactive lymphocytes [34,35,36]. In addition, immunosuppressive drugs that are used to treat AAV could either indirectly affect the immune response by hampering the clearance of cancer cells under homeostatic conditions or directly promote carcinogenesis. These hypotheses are supported by the results from previous studies that have demonstrated that immunocompromised individuals, such as organ recipients and patients with human immunodeficiency virus infections, are at higher risk of developing cancers [37,38]. In addition, compelling experimental evidence suggests that chronic exposure to immunomodulatory drugs may accelerate or initiate the process of carcinogenesis [39,40].

To identify the incidence of de novo cancers in patients with AAV, we excluded patients with a history of cancer before the index date of AAV. Surprisingly, we found that a considerable number of patients (*n* = 328) were excluded due to their history of cancer upon patient selection. Furthermore, the mean follow-up duration of patients with cancer was 1.5 years, which was relatively short. These findings appear to be in accordance with the results in previous studies, which showed that the incidence of cancer was most prominent during the period of diagnosis in patients with inflammatory myositis [41,42]. However, we were not able to calculate the SIRs of cancers prior to the diagnosis of AAV because the obtained data were not indicative of the exact period of the diagnosis of cancer before the onset of AAV. In addition, because a recent study has also suggested that the risk of malignancy prior to the diagnosis of AAV was not elevated, future studies are required to elucidate whether preceding cancer can directly promote the development of AAV [43].

In this study, we found a significantly heightened incidence of overall cancer in AAV patients. Meanwhile, in a subgroup analysis based on sex and the subtypes of AAV, a different pattern of cancer incidence was found. Briefly, the risks of gallbladder, lung, and hematologic cancers were higher in males, whereas the risks of bladder, hematologic cancer, and the remaining cancers were higher in females. However, the higher risk of NMSC in AAV, which was found to be the most prominent site-specific cancer in the Western world, was not observed herein. In addition, among subgroups of AAV, the SIRs for overall cancer were the highest in subjects with GPA, which is consistent with a previous report [18]. In particular, the higher risk of lung cancer in GPA seems to be convincing because a common clinical feature of GPA is the involvement of the respiratory tracts [44]. Notably, patients with EGPA were also at increased risk of cancers, with a similar incidence pattern of site-specific cancers except lung cancer, compared to GPA. These findings imply the need for a different approach with regard to the type of cancer depending on sex and the subtypes of AAV, as well as the geographic location.

Previous studies have reported that the administration of immunosuppressive agents is associated with the incidence of cancers. It has been demonstrated that methotrexate and azathioprine, which are widely used drugs for the treatment of AIDs, confer a higher risk of cancers [45,46]. Our data demonstrated that all treatments except rituximab were associated with an increased risk of overall cancer. This finding is in line with that of a recent publication by van Daalen et al. [47] in which rituximab treatment for AAV was not associated with the risk of cancer compared to the general population. On the other hand, although it has been suggested that the use of cyclophosphamide was associated with the risk of bladder cancer [48], the risk of bladder cancer was not elevated in this study. This non-significant association seems to be related to the lower cumulative dose of cyclophosphamide used, as cyclophosphamide is now usually used to induce remission in AAV. Accordingly, the cyclophosphamide dose used for AAV may not be sufficiently high to induce carcinogenesis in the bladder [49]. However, because patients treated with immunosuppressive agents generally had a higher risk of cancers and a distinct pattern of site-specific cancer incidence, it is essential to find measures to optimize the control of the disease while minimizing the use of immunosuppressants. This is further emphasized by the fact that the administration of cyclophosphamide and methotrexate and longer usage of glucocorticoids were independent factors associated with the development of cancer in a nested case-control study among patients with AAV.

Age is an important factor related to the development of cancer in the general population [50]. Notably, in the present study, an increased risk of cancer was found in AAV patients across all age groups with the only exception in those ≥ 80 years old. In addition, a similar trend was found in males, with a tendency towards increased SIRs with decreasing age. Interestingly, AAV patients who were <50 years and 50–59 years old had significantly higher SIRs for overall and hematological cancers compared to the other age groups. Given that younger subjects are less likely to be affected by cancer, these results suggest that special attention and active measures to detect the occurrence of cancers (e.g., routine health screening programs) should be provided even in younger AAV subjects, particularly in those <60 years old.

In this study, the proportion of patients with EGPA was 27.7%, which was higher compared to the proportion of patients with EGPA among AAV in the reports from Japan [51,52]. Even though the exact cause of this finding is unclear, difference in the methods applied (ICD-10 codes were used to extract patient data in our study and 2007 European Medicines Agency criteria were applied to those with the diagnosis of AAV in the study from Japan) and the inclusion of renal-limited and unclassified vasculitis could have resulted in the discrepant results. However, recent publication from our group using a tertiary hospital data revealed that the proportion of patients with EGPA was similar to the present study [53], also suggesting that a regional difference could be present.

The main strength of this study is that this is the first study to assess the risk of cancer in patients with AAV in Asia by using a database that encompasses the information for all healthcare beneficiaries in a single country. In addition, the number of patients included is the largest thus far in studies assessing the risk of cancer in patients with AAV. However, there are several limitations in our study. First, the exact number of patients with AAV may have been underestimated. Because we have defined our patients as having AAV based on ICD-10 codes and the use of glucocorticoids, patients who were not treated for AAV and those who received medical treatment without glucocorticoids may have been excluded from the study. Moreover, patients with renal-limited vasculitis and unclassified vasculitis could not be searched because the ICD-10 codes are not available for these diseases, which could have also influenced the result of our study. Second, conventional risk factors for cancer in the general population, such as alcohol consumption, smoking (especially for lung cancer), dietary habits, complications, and genetic factors could not be investigated, and direct interpretation of the results of this study should be made with caution [54]. Third, more detailed AAV-specific variables, including ANCA serotypes, disease activity, and the associations between organ-specific involvement and cancers, were not obtainable. Fourth, the cumulative dose of immunosuppressive agents related to cancer incidence was not calculable. Finally, the use of calcineurin inhibitors (tacrolimus and cyclosporine) and intravenous immunoglobulin therapy could not be investigated because the use of either agent is not currently covered by the NHI and is not recorded in the HIRA database.

## 5. Conclusions

In summary, we have demonstrated that compared to the general population, AAV patients have an increased overall risk of cancer and increased risks of site-specific lung and hematological cancers. In addition, different patterns of cancer incidence were found according to age, sex, AAV subtypes, and the usage of immunosuppressive agents. The findings from our study imply that regular monitoring of cancers and efforts to minimize the administration of immunosuppressive agents in the treatment of AAV are necessary.

## Figures and Tables

**Table 1:** Comparison of clinical characteristics between AAV Patients with and without cancer.

	Total *n* = 2097	AAV Patients with Cancer *n* = 114	AAV Patients without Cancer *n* = 1983	*p*-Value
Age at diagnosis	59.8 ± 15.6	61.7 ± 13.9	59.7 ± 15.7	0.1723
Sex, *n* (%)				
Female	1168 (55.7)	53 (46.5)	1115 (56.2)	0.0526
Male	929 (44.3)	61 (53.5)	868 (43.8)	
Diagnosis, *n* (%)				
MPA	947 (45.2)	38 (33.3)	909 (45.8)	0.0004
GPA	568 (27.1)	49 (43.0)	519 (26.2)	
EGPA	582 (27.7)	27 (23.7)	555 (28.0)	
Type of insurance, *n* (%)				
National Health Insurance	2004 (95.6)	109 (95.6)	1895 (95.6)	1.0000
Medical Aid	93 (4.4)	5 (4.4)	88 (4.4)	
CCI subcategories, *n* (%) ^a^				
Cardiovascular disorder				
Myocardial infarction	53 (2.5)	2 (1.8)	51 (2.6)	1.0000
Congestive heart failure	208 (9.9)	16 (14.0)	192 (9.7)	0.1767
Peripheral vascular disease	389 (18.6)	19 (16.7)	370 (18.7)	0.6831
Cerebrovascular disease	303 (14.5)	11 (9.7)	292 (14.7)	0.1732
Diabetes	702 (33.5)	36 (31.6)	666 (33.6)	0.7343
Diabetes with chronic complication	240 (11.4)	11 (9.7)	229 (11.6)	0.6397
Gastrointestinal disorder				
Mild liver disease	769 (36.7)	36 (31.6)	733 (37.0)	0.2890
Moderate or severe liver disease	12 (0.6)	1 (0.9)	11 (0.6)	0.4896
Peptic ulcer disease	840 (40.1)	41 (36.0)	799 (40.3)	0.4130
Pulmonary disorder				
Chronic pulmonary disease	1420 (67.7)	64 (56.1)	1356 (68.4)	0.0089
Rheumatologic disorder				
Rheumatologic disease	330 (15.7)	15 (13.2)	315 (15.9)	0.5187
Kidney disorder				
Renal disease except unspecified kidney failure	373 (17.8)	14 (12.3)	359 (18.1)	0.1456
Others				
Dementia	86 (4.1)	2 (1.8)	84 (4.2)	0.3247
Hemiplegia or paraplegia	48 (2.3)	1 (0.9)	47 (2.4)	0.5159
Malignancy (nonmetastatic cancer)	0 (0.0)	0 (0.0)	0 (0.0)	*n*/*a*
Metastatic solid tumor	0 (0.0)	0 (0.0)	0 (0.0)	*n*/*a*
Acquired immune deficiency syndrome	0 (0.0)	0 (0.0)	0 (0.0)	*n*/*a*
Medication usage, *n* (%)				
Glucocorticoid steroid usage ≥ 1 year	1037 (49.5)	67 (58.8)	970 (48.9)	0.0511
Cyclophosphamide	1022 (48.7)	63 (55.3)	959 (48.4)	0.1811
Rituximab	250 (11.9)	8 (7.0)	242 (12.2)	0.1303
Azathioprine/mizoribine	835 (39.8)	48 (42.1)	787 (39.7)	0.6785
Methotrexate	293 (14.0)	25 (21.9)	268 (13.5)	0.0173

^a^ The medical conditions included in the CCI score were investigated within one year of the AAV index date. Values are expressed as the mean ± standard deviation or as the number (percentage). AAV: ANCA-associated vasculitis; ANCA: Antineutrophil cytoplasmic antibody; MPA: Microscopic polyangiitis; GPA: Granulomatosis with polyangiitis; EGPA: Eosinophilic granulomatosis with polyangiitis; CCI: Charlson Comorbidity Index; n/a: Not applicable.

**Table 2:** Estimation of cancer risk in AAV patients.

Cancer (ICD-10)	Total			
	Expected	Observed	SIR	95% CI
Overall cancer (C00–C96)	60.06	114	1.90	(1.57–2.28)
Stomach (C16)	9.12	9	0.99	(0.45–1.87)
Colon and rectum (C18–C20)	8.41	9	1.07	(0.49–2.03)
Liver (C22)	4.83	4	0.83	(0.23–2.12)
Gallbladder, etc. (C23–C24)	1.97	5	2.54	(0.82–5.93)
Pancreas (C25)	2.03	3	1.48	(0.30–4.32)
Lung (C33–C34)	8.51	19	2.23	(1.34–3.48)
Breast (C50)	3.28	3	0.91	(0.19–2.67)
Ovary (C56)	0.50	2	3.98	(0.48–14.39)
Prostate (C61)	3.84	1	0.26	(0.01–1.45)
Kidney (C64)	1.18	4	3.40	(0.93–8.70)
Bladder (C67)	1.36	4	2.94	(0.80–7.54)
Thyroid (C73)	4.65	2	0.43	(0.05–1.55)
Hematological cancer	2.28	26	11.39	(7.44–16.69)
Hodgkin lymphoma (C81)	0.05	1	21.85	(0.55–121.75)
Non-Hodgkin lymphoma (C82–C85, C96)	1.28	13	10.14	(5.40–17.34)
Multiple myeloma (C90)	0.47	10	21.12	(10.13–38.85)
Leukemia (C91–C95)	0.48	2	4.16	(0.50–15.01)
Other (remaining cancer codes)	7.40	23	3.11	(1.97–4.66)

AAV: ANCA-associated vasculitis; ANCA: Antineutrophil cytoplasmic antibody; ICD: International classification of diseases.

**Table 3:** Estimation of cancer risk according to sex in AAV.

Cancer (ICD-10)	Male				Female			
	Expected	Observed	SIR	95% CI	Expected	Observed	SIR	95% CI
Overall cancer (C00–C96)	34.96	61	1.74	(1.33–2.24)	25.79	53	2.06	(1.54–2.69)
Stomach (C16)	6.10	5	0.82	(0.27–1.91)	2.93	4	1.36	(0.37–3.49)
Colon and rectum (C18–C20)	5.00	7	1.40	(0.56–2.88)	3.38	2	0.59	(0.07–2.14)
Liver (C22)	3.35	3	0.89	(0.18–2.62)	1.38	1	0.73	(0.02–4.04)
Gallbladder, etc. (C23–C24)	1.05	4	3.82	(1.04–9.78)	0.92	1	1.08	(0.03–6.04)
Pancreas (C25)	1.08	1	0.93	(0.02–5.17)	0.95	2	2.12	(0.26–7.64)
Lung (C33–C34)	6.29	14	2.23	(1.22–3.74)	2.38	5	2.10	(0.68–4.90)
Breast (C50)					3.57	3	0.84	(0.17–2.46)
Ovary (C56)					0.53	2	3.75	(0.45–13.54)
Prostate (C61)	3.88	1	0.26	(0.01–1.44)				
Kidney (C64)	0.77	3	3.90	(0.80–11.40)	0.39	1	2.57	(0.06–14.31)
Bladder (C670	1.14	1	0.88	(0.02–4.90)	0.25	3	12.05	(2.48–35.21)
Thyroid (C73)	0.84	1	1.19	(0.03–6.63)	4.13	1	0.24	(0.01–1.35)
Hematological cancer	1.25	12	9.59	(4.96–16.76)	1.03	14	13.58	(7.42–22.78)
Hodgkin lymphoma (C81)					0.02	1	48.19	(1.22–268.50)
Non-Hodgkin lymphoma (C82–C85, C96)	0.70	6	8.60	(3.16–18.72)	0.59	7	11.95	(4.81–24.63)
Multiple myeloma (C90)	0.25	5	20.01	(6.50–46.70)	0.22	5	22.30	(7.24–52.04)
Leukemia (C91–C95)	0.28	1	3.58	(0.09–19.93)	0.20	1	4.99	(0.13–27.81)
Other (remaining cancer codes)	4.19	9	2.15	(0.98–4.08)	3.23	14	4.34	(2.37–7.28)

AAV: ANCA-associated vasculitis; ANCA: Antineutrophil cytoplasmic antibody; ICD: International classification of diseases.

**Table 4:** Cancer-specific risk based on subgroups of AAV.

Cancer (ICD-10)	MPA				GPA				EGPA			
	Expected	Observed	SIR	95% CI	Expected	Observed	SIR	95% CI	Expected	Observed	SIR	95% CI
Overall cancer (C00–C96)	26.89	38	1.41	(1.00–1.94)	17.19	49	2.85	(2.11–3.77)	15.98	27	1.69	(1.11–2.46)
Stomach (C16)	4.16	5	1.20	(0.39–2.81)	2.61	2	0.77	(0.09–2.77)	2.36	2	0.85	(0.10–3.07)
Colon and rectum (C18–C20)	3.87	4	1.03	(0.28–2.65)	2.40	5	2.08	(0.68–4.86)	–			
Liver (C22)	2.18	3	1.38	(0.28–4.03)	1.39	1	0.72	(0.02–4.02)	–			
Gallbladder, etc. (C23–C24)	0.95	2	2.10	(0.25–7.60)	0.55	1	1.80	(0.05–10.05)	0.46	2	4.32	(0.52–15.60)
Pancreas (C25)	0.97	2	2.07	(0.25–7.48)	0.57	1	1.74	(0.04–9.70)	–			
Lung (C33–C34)	4.09	7	1.71	(0.69–3.53)	2.40	10	4.17	(2.00–7.66)	2.03	2	0.99	(0.12–3.56)
Breast (C50)	–				0.97	1	1.03	(0.03–5.74)	1.13	2	1.78	(0.22–6.42)
Ovary (C56)	–				0.15	1	6.83	(0.17–38.05)	0.15	1	6.55	(0.17–36.50)
Prostate (C61)	–				–				0.89	1	1.12	(0.03–6.25)
Kidney (C64)	0.51	2	3.92	(0.47–14.15)	0.34	1	2.93	(0.07–16.31)	0.33	1	3.07	(0.08–17.12)
Bladder (C67)	0.65	3	4.62	(0.95–13.51)	–				0.33	1	3.07	(0.08–17.10)
Thyroid (C73)	–				1.38	1	0.72	(0.02–4.02)	1.68	1	0.59	(0.02–3.31)
Hematological cancer	1.02	6	5.86	(2.15–12.75)	0.65	12	18.40	(9.51–32.15)	0.61	8	13.20	(5.70–26.01)
Hodgkin lymphoma (C81)	–				–				0.01	1	72.78	(1.84–405.58)
Non-Hodgkin lymphoma (C82–C85, C96)	0.57	1	1.75	(0.04–9.75)	0.37	7	19.08	(7.67–39.31)	0.34	5	14.56	(4.73–33.99)
Multiple myeloma (C90)	0.22	5	22.54	(7.32–52.59)	0.13	3	22.29	(4.60–65.15)	0.12	2	17.10	(2.07–61.78)
Leukemia (C91–C95)	–				0.14	2	14.52	(1.76–52.47)	–			
Other (remaining cancer codes)	3.37	4	1.19	(0.32–3.03)	2.11	13	6.15	(3.27–10.51)	1.91	6	3.13	(1.15–6.82)

AAV: ANCA-associated vasculitis; ANCA: Antineutrophil cytoplasmic antibody; ICD: International classification of diseases; MPA: Microscopic polyangiitis; GPA: Granulomatosis with polyangiitis; EGPA: Eosinophilic granulomatosis with polyangiitis.

**Table 5:** Factors associated with the development of cancer among patients with AAV.

	Crude Odds Ratio ^a^			Adjusted Odds Ratio		
	Odds Ratio	95% CI	*p*-Value	Odds Ratio	95% CI	*p*-Value
Type of insurance						
National Health Insurance	1.0 (ref)			1.0 (ref)		
Medical Aid	1.11	(0.38–3.28)	0.8508	1.47	(0.47–4.57)	0.5092
Glucocorticoid usage ≥ 1 year	4.36	(1.61–11.84)	0.0039	4.20	(1.47–12.01)	0.0075
Cyclophosphamide	1.78	(1.08–2.93)	0.0239	1.75	(1.03–2.96)	0.0385
Rituximab	0.78	(0.24–2.49)	0.6731	0.56	(0.17–1.92)	0.3591
Azathioprine/mizoribine	1.33	(0.77–2.30)	0.3087	0.94	(0.50–1.75)	0.8347
Methotrexate	2.27	(1.11–4.63)	0.0247	2.39	(1.12–5.13)	0.0247

^a^ In the nested case-control analysis, the selected cases were matched for sex, age, AAV index date, duration, and type of diagnosis. AAV: ANCA-associated vasculitis; ANCA: Antineutrophil cytoplasmic antibody.

## Supplementary Materials

The following are available online at https://www.mdpi.com/2077-0383/8/11/1871/s1, Supplementary Figure S1. Flowchart for patient selection; Supplementary Table S1. Cancer-specific risk based on the use of cyclophosphamide and rituximab in patients with AAV; Supplementary Table S2. Cancer-specific risk based on the use of azathioprine/mizoribine and methotrexate in patients with AAV.
