# Supplementary material for: Risk of Cancers in Antineutrophil Cytoplasmic Antibody-Associated Vasculitis: Results from the Korea National Health Insurance Claims Database 2010–2018
Source: J Clin Med. 2019 Nov 5;8(11):1871. doi: 10.3390/jcm8111871 (PMC6912195; doi:10.3390/jcm8111871)
Supplement: Supplementary file 1 [file jcm-08-01871-s001.pdf]

Figure S1. Flowchart for patient selection.

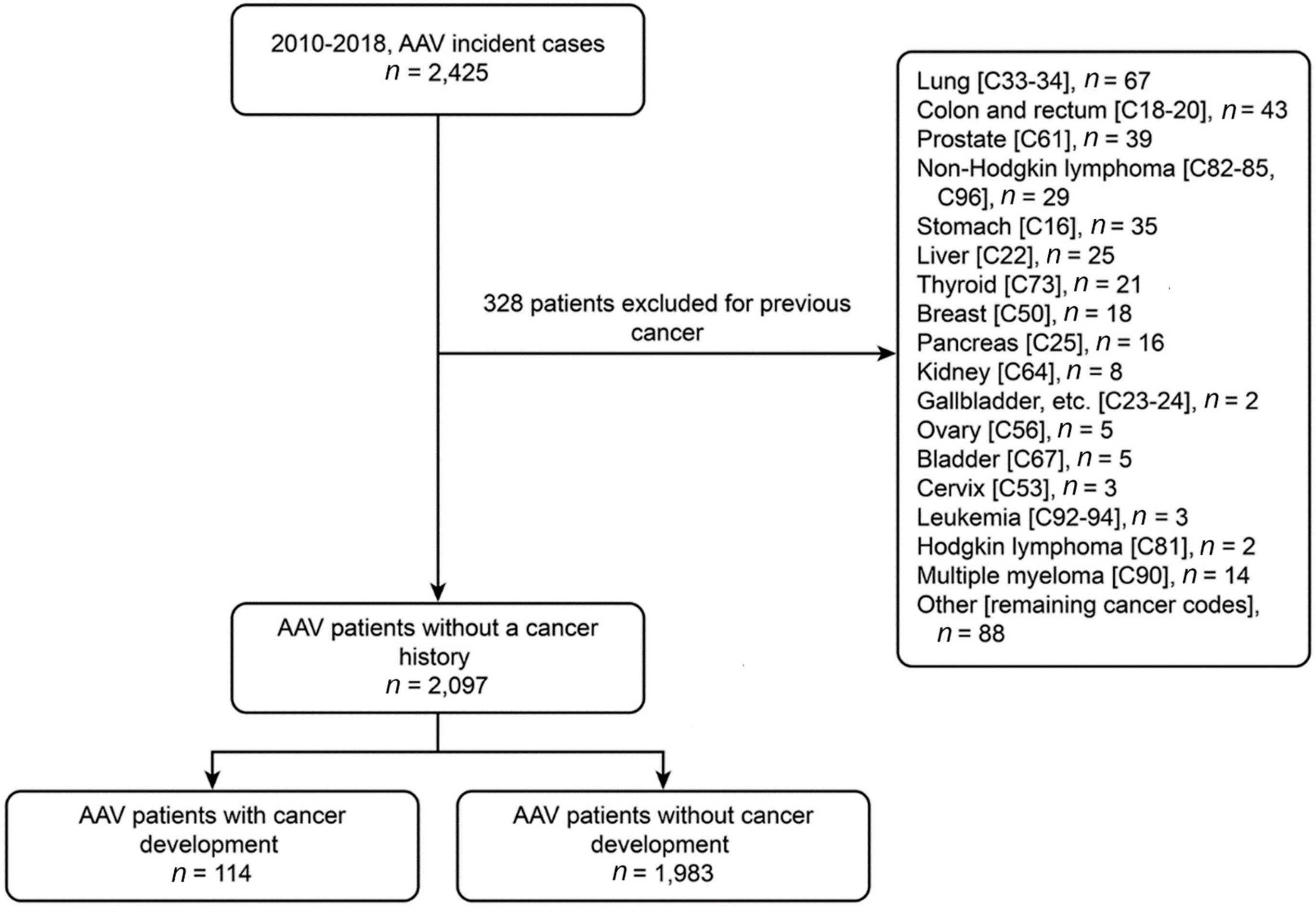

Table S1. Cancer-specific risk based on the use of cyclophosphamide and rituximab in patients with AAV.

Cancer (ICD–10)	Cyclophosphamide				Rituximab			
	**Expected**	**Observed**	**SIR**	**95% CI**	**Expected**	**Observed**	**SIR**	**95% CI**
Overall cancer (C00–C96)	30.28	63	**2.08**	**(1.60–2.66)**	6.41	8	1.25	(0.54–2.46)
Stomach (C16)	4.64	7	1.51	(0.61–3.11)	–			
Colon and rectum (C18–C20)	4.26	3	0.70	(0.15–2.06)	0.89	1	1.12	(0.03–6.23)
Liver (C22)	2.46	3	1.22	(0.25–3.57)	–			
Gallbladder, etc. (C23–C24)	0.98	3	3.07	(0.63–8.97)	–			
Pancreas (C25)	–				–			
Lung (C33–C34)	4.31	10	**2.32**	**(1.11–4.27)**	–			
Breast (C50)	1.63	2	1.23	(0.15–4.43)	–			
Ovary (C56)	0.25	1	4.03	(0.10–22.44)	–			
Prostate (C61)	–				–			
Kidney (C64)	0.60	3	**5.01**	**(1.03–14.63)**	0.13	2	**15.39**	**(1.86–55.61)**
Bladder (C67)	0.68	2	2.94	(0.36–10.63)	–			
Thyroid (C73)	–				–			
Hematological cancer	1.16	16	**13.82**	**(7.90–22.45)**	0.25	2	8.13	(0.98–29.37)
Hodgkin lymphoma (C81)	–				–			
Non–Hodgkin lymphoma (C82–C85, C96)	0.65	10	**15.40**	**(7.38–28.32)**	0.14	2	**14.47**	**(1.75–52.26)**
Multiple myeloma (C90)	0.24	5	**20.63**	**(6.70–48.13)**	–			
Leukemia (C91–C95)	0.24	1	4.11	(0.10–22.91)	–			
Other (remaining cancer codes)	3.71	13	**3.51**	**(1.87–6.00)**	0.78	3	3.83	(0.79–11.21)

AAV: ANCA-associated vasculitis; ANCA: Antineutrophil cytoplasmic antibody; ICD: International classification of diseases.

Table S2. Cancer-specific risk based on the use of azathioprine/mizoribine and methotrexate in patients with AAV

Cancer (ICD-10)	Azathioprine/mizoribine				Methotrexate			
	**Expected**	**Observed**	**SIR**	**95% CI**	**Expected**	**Observed**	**SIR**	**95% CI**
Overall cancer (C00–C96)	27.70	48	**1.73**	**(1.28–2.30)**	8.88	25	**2.82**	**(1.82–4.16)**
Stomach (C16)	4.25	5	1.18	(0.38–2.75)	1.34	4	3.00	(0.82–7.67)
Colon and rectum (C18–C20)	3.89	3	0.77	(0.16–2.25)	1.22	1	0.82	(0.02–4.59)
Liver (C22)	2.24	3	1.34	(0.28–3.91)	0.72	1	1.39	(0.04–7.75)
Gallbladder, etc. (C23–C24)	0.89	3	3.38	(0.70–9.87)	–			
Pancreas (C25)	0.93	2	2.16	(0.26–7.81)	–			
Lung (C33–C34)	3.92	7	1.79	(0.72–3.68)	1.16	2	1.73	(0.21–6.25)
Breast (C50)	–				0.57	1	1.75	(0.04–9.75)
Ovary (C56)	0.23	1	4.37	(0.11–24.32)	–			
Prostate (C61)	–				–			
Kidney (C64)	0.55	3	**5.46**	**(1.13–15.96)**	0.18	1	5.48	(0.14–30.51)
Bladder (C67)	0.62	2	3.22	(0.39–11.65)	–			
Thyroid (C73)	2.12	2	0.94	(0.11–3.41)	0.86	1	1.17	(0.03–6.49)
Hematological cancer	1.06	6	**5.67**	**(2.08–12.35)**	0.34	6	**17.72**	**(6.50–38.57)**
Hodgkin lymphoma (C81(	–				–			
Non–Hodgkin lymphoma (C82–C85, C96)	0.59	3	**5.06**	**(1.04–14.78)**	0.19	6	**31.34**	**(11.50–68.22)**
Multiple myeloma (C90)	0.22	2	**9.03**	**(1.09–32.61)**	–			
Leukemia (C91–C95)	0.22	1	4.52	(0.11–25.18)	–			
Other (remaining cancer codes)	3.38	11	**3.25**	**(1.62–5.81)**	1.08	8	**7.44**	**(3.21–14.66)**

AAV: ANCA-associated vasculitis; ANCA: Antineutrophil cytoplasmic antibody; ICD: International classification of diseases.
